# Analysis of facial emotion expression in eating occasions using deep learning

**DOI:** 10.1007/s11042-023-15008-6

**Published:** 2023-03-22

**Authors:** Elif Yildirim, Fatma Patlar Akbulut, Cagatay Catal

**Affiliations:** 1 Department of Computer Engineering, Istanbul Kültür University, Istanbul, Turkey; 2 Department of Computer Science and Engineering, Qatar University, Doha, Qatar

**Keywords:** Emotion recognition, Affective computing, Food, Deep learning

## Abstract

Eating is experienced as an emotional social activity in any culture. There are factors that influence the emotions felt during food consumption. The emotion felt while eating has a significant impact on our lives and affects different health conditions such as obesity. In addition, investigating the emotion during food consumption is considered a multidisciplinary problem ranging from neuroscience to anatomy. In this study, we focus on evaluating the emotional experience of different participants during eating activities and aim to analyze them automatically using deep learning models. We propose a facial expression-based prediction model to eliminate user bias in questionnaire-based assessment systems and to minimize false entries to the system. We measured the neural, behavioral, and physical manifestations of emotions with a mobile app and recognize emotional experiences from facial expressions. In this research, we used three different situations to test whether there could be any factor other than the food that could affect a person’s mood. We asked users to watch videos, listen to music or do nothing while eating. This way we found out that not only food but also external factors play a role in emotional change. We employed three Convolutional Neural Network (CNN) architectures, fine-tuned VGG16, and Deepface to recognize emotional responses during eating. The experimental results demonstrated that the fine-tuned VGG16 provides remarkable results with an overall accuracy of 77.68% for recognizing the four emotions. This system is an alternative to today’s survey-based restaurant and food evaluation systems.

## Introduction

Emotions are complex psycho-physiological change arising from the interaction of an individual with biochemical [8] and environmental [32] influences. It is the main factor that determines the individual sense of health and plays a central role in the daily life of the person. There are numerous types of emotions that have an impact on how people live and are associated with other people [14]. Sometimes, people are dominated by these emotions. The choices they make, the activities they take, and the recognition they have are all impacted by the emotions they encounter at any given time [25]. Besides, emotions can be also affected by many things [20], such as daily activities, the places visited, and the people spent time together. The foods have a significant effect on the emotions as well [30]. Recent research findings suggest that the food that is consumed acts as a mood regulator [11] in both positive and negative ways. People have a complex relationship with food. When people are happy, sad, or feel other emotions, they choose food based on the emotion they feel. Food is present in almost every aspect of people’s lives and can affect their mood significantly.

A clear understanding of customers’ emotions can provide various benefits to companies and organizations such as improving customer satisfaction, establishing the connection between the customer and brand, and consequently, commercial success. Moreover, understanding the affective behavior of humans can help product managers to better understand to identify the products with the highest impact. According to TripAdvisor’s survey [37], 94% of diners choose their restaurants based on online reviews. The reason why review sites are taken as a reference is that there are many restaurants to choose from and the customer does not have the opportunity and time to experience all of them individually. In addition, it is important to get benefits from the best practices and experiences. Today, sites such as Yelp, TripAdvisor, Zomato, and OpenTable are global priority channels where restaurants and dining experiences are shared and evaluated. Not all comments are reliable on these platforms. Therefore, these platforms also include reviews from more trusted people, such as food bloggers. They seek different ways to reduce the bias in the evaluation of the eating experience.

Today’s world benefits from automatic emotion recognition systems in different domains [6] such as patient care, medical diagnosis, education, video gaming, automotive assistance, recruiting personnel for companies, fraud detection for finance, etc.. For instance, emotion recognition systems can play a significant role in healthcare because the patient’s diagnosis and the recovery period can be understood and managed with higher accuracy [1]. In addition, it is utilized in tough follow-up processes of depression and anxiety patients [2]. Another effective utilization area is the evaluation of the student/lecturer interaction [31] during online education. Especially, during the Covid-19 pandemic, online education has increased dramatically all around the world, therefore, it needs to be supported with innovative approaches more than ever. The literature contains plenty of studies that aim to detect only emotions [3, 29] with similar approaches to this study. As valuable examples, Vatcharaphrueksadee et al. [39] and Jaiswal et al. [21] work with facial data and use CNN models to classify emotions. However, the aim of these studies is to develop a generic emotion recognition system that can be used in the above-mentioned domains. To the best of our knowledge, this is the first attempt to detect emotion during eating occasions.

In literature, even if there are a lot of studies investigating the relationship between eating and emotions, most of them contain questionnaire-based survey data. The aim of this study is to detect emotions in real time. Despite the studies’ work on the same subject with continuous or real-time data being rare, few examples can be found in the state of the art. An example of this is the work of Carroll et al. [10], which performs emotion recognition by using ECG and EDA signals. In the study, the effects of emotional eating behavior in humans through physiological signals were investigated.

To elicit emotional eating, successful sentimental stimulation must be experienced, which may include a person’s reaction to the meals. For this reason, we avoided triggering the emotion with an external method. We asked the individual to convey what they felt at the time of eating so that the precise causal effect of emotions on food intake could be determined. We examined certain emotions from the consumer perspective and a variety of models were put forward. In literature, studies are mostly clustered around the six basic emotions, namely happiness, fear, sadness, surprise, anger, and disgust, although they were expanded later on. Hence, we planned to analyze six basic emotions, however, three of these emotions, namely happy, sad, and disgusted and, neutral status were experienced during the experiments. Particularly, we aim to analyze how food affects emotions by examining facial expressions. To achieve this objective, an emotion recognition model was developed using state-of-the-art deep learning approaches. In our proposed approach, we developed a mobile application that users can capture videos of themselves while eating. We built a fine-tuned VGG16 model to recognize the emotional status of participants on eating occasions. We also use the DeepFace algorithm [36] that a nine-layer neural network with over 120 million connections and is able to capture demographic information such as the age, race, gender, and emotion of that person from the facial features to validate our prediction model in real-time. Our contributions are three-fold:

- We developed a system that recognizes emotions during eating occasions.
- We evaluated the performance of different deep learning-based algorithms for this problem.
- We demonstrated the effectiveness of the fine-tuned deep learning-based emotional status prediction system.

This paper is structured as follows: Section 2 explains the related work. Section 3 describes the method used in this study. Section 4 presents the experimental results. Section 5 discusses the conclusion.

## Related work

Motivation is one of the fundamental forces driving behavior. An person’s desire to eat is a biological source of motivation. According to the two-factor theory of emotion, [33], the experience of emotion is regulated by the strength of the experienced arousal, while the intensity of the desire to eat determined what the emotion and intensity will be. Various studies aimed to model this bond. Carroll et al. [10] aimed to discourage people from emotional eating habit. According to their study, people are more prone to emotional eating when they are distressed or upset. By detecting the negative emotions just before the eating with a wearable system, it is possible to help people to change their unhealthy eating patterns. In a similar study by King and Meiselman [23], it was mentioned how positive emotions are particularly effective on people with obesity problems. In the study, it was concluded that stress increases the level of eating, and positive emotions balance this level a little bit, even more, effective than a diet.

Based on previous research, we can say that emotions and food have a direct relationship. Kenney and Adhikari’s study [22] revealed that emotion is the essential factor when it comes to conceptualizing the customer experience and predicting the demand of different customer profiles in the food industry. In another research, the results indicate that food flavors are powerful clues that activate memories and stimulate instinctive responses. This way, a considerable amount of the food servicing sector takes advantage of natural product flavors to tempt consumers. For instance, most donut chains [28], places its bakeries at the store entrance and are spread around with the seductive scent of product to entice clients. In addition, some cafeterias encourage clients to buy more by adding delightful flavors to their dining rooms. An experiment [34] in the deli revealed that after the introduction of coffee flavors into the setting, beverage sales increased 300%. Also, the research has revealed that aroma is an important factor affecting client fulfillment and attitude, in conformity with the restaurant atmosphere model. Frayn et al. [15] reported that female students having sentimental feeding accrued foods consumption that contain added sugar following a negative state of mind induction, though no increase was reported when salty foods are consumed. In another article by van Strien et al. [38], the difference between two female groups, which are highly emotional eaters and low emotional eaters, had been investigated. It was found that the group that consisted of low emotional eaters ate a similar amount of food after joy and sadness, while the high emotional eaters’ group ate more food when they were unhappy than when they were happy.

The foods that affect emotions, mood, and the social environment have also an effect on choosing the type and the amount of food consumed. There are situations where roles change during the relationship between eating and emotions. Just like the foods that affect emotions, mood and the social environment have also an effect on choosing the type and the amount of food consumed. For instance, a person can eat a dessert when he feels unhappy, or he can enjoy the meal more when he is happy. Studies have shown that the social environment increases eating. It can be said that the rate of eating at a wedding or a school cafeteria is high. The reason is that a joyful environment such as a wedding or a meal eaten by chatting with friends is more relaxing and creates a desire to eat more because it increases the happiness hormone. Eating tempting meals while in a positive mood can stimulate appetite and people can eat more [13]. Moreover, in a study of people having eating disorders (i.e., bulimia nervosa) [9], people with this ailment watched positive and neutral video clips, respectively, to determine whether people can have a positive mood. Thanks to positive video clips, it has been observed that people with these two different ailments have more regular levels of eating.

We observed that most of the studies conducted to reveal the relationship between emotions and food use observation and survey techniques. As shown on Table 1, Ouyang et al. [28] , Frayn et al. [15], van Strien et al. [38], Evers et al. [13], Cardi et al. [9], Altheimer et al. [4], and Herren et al. [19] used survey data. The highlight of our study is that the resultant data is supported by a piece of physical evidence that is taken instantly by the mobile application.

**Table 1:** Comparative table between the related works

Study	The goal	Techniques used	Dataset employed	Sample size(N)	Evaluation measures
Carroll et al. [10]	To prevent emotional eating	Normalization, feature extraction, Gaussian Process Regression	EKG and EDA signals	12	Valence= 72.62%, Arousal= 75.00%
Ouyang et al. [28]	food aromas and consumer emotions relationship	Logistic Regression	Survey	196	B(Wald)= 0.386, Exp(B)= 1.472, p-value= 0.137
Frayn et al. [15]	To find the food addiction after sad mood induction	Eye Tracking with EyeLink sensor	Survey and fixation data	66	Emotional Eating
van Strien et al. [38]	emotional eating and food intake relationship in sadness and joy	VR–MIP, ANOVA	Survey	60	Dutch Eating Behavior Questionnaire
Evers et al. [13]	observing positive emotions as a trigger for food intake	ANOVA	Survey	S1: 68, S2: 84	F(1, 37)= 60.40, p < 0.001, pn(eta)ˆ2 = 0.62
Cardi et al. [9]	observing positive emotions to reduce the overeating behaviour	ANOVA	Survey	30	p = 0.002
Vatc. et al. [39]	Facial emotion recognition	CNN	CK+, FER2013	123, 123	65%
Jaiswal et al. [21]	Facial emotion recognition	CNN	FERC2013, JAFFE	123, 10	FERC2013 = 70.14%, JAFFE= 98.65%
Altheimer et al. [4]	relation of sadness and anxiety to emotional eating	Bivariate correlation, LMER	Survey	S1: 118, S2: 111	DEQ, PSS, DSI
Herren et al. [19]	To find if ES is associated with BMI through EE, and the role of physical activities, ethnicity and gender	Linear Regression	Survey	1674	B = 0.0017, CI 95% [0.0001, 0.0042]
Our study	To detect emotions while eating	CNN, Fine tuned VGG-16	FER-2013, CK+, Tailored Dataset	123, 123, 13	77.68%

Even if people are largely aware of the emotions they are experiencing, they may mislabel the source of the arousal or wish to deceive the system. One of the ways to overcome this is to understand emotions from non-verbal behaviors and express them to others in this way. This is where the role of understanding emotions by analyzing facial expressions or physiological signals comes into play like Carroll et al.’s [10] study. They use physiological signals (ECG and EDA) in their study, and the accuracies they get from their model are 72% and 75% which is close to the accuracy we get from our models, but it is hard to compare the two works because the type of data used is different from each other. Vatcharaphrueksadee et al. [39] developed a VGG-16-based model with CK+ 48 and FER2013 datasets and reached 65% accuracy. We also train models with the same approach and get an accuracy of 77% from fine-tuned VGG-16 model. Jaiswal et al. [21] get 70% accuracy from their CNN model, using the FERC2013 dataset, which is a blurry version of FER2013 data.

The conclusion from most studies is that the idea that certain foods can have habit-forming effects has acquired a lot of awareness over the last years. Many studies showed that foods rich in sugar and fat affect the reward systems in the brain. Studies [16] established that both fat and sugar consumption releases the dopamine and provides delightful effects. Many people turn to food to reduce negative feeling, and a few analysis has supported this linkage.

## Method

We conducted a user study to capture the emotional changes while eating through facial expressions and used videos taken during meals to analyze people’s emotions. We aimed for individuals to record their own emotions in order not to experience emotional changes caused by third parties around them. In this context, a mobile application was developed and a system was introduced for participants to record themselves while eating. This way users can instantly forget that they are in an experiment and perform their eating activities. The following subsections provide more information about the method applied in this research.

### Development of a mobile application to capture emotions

**Fig. 1:**
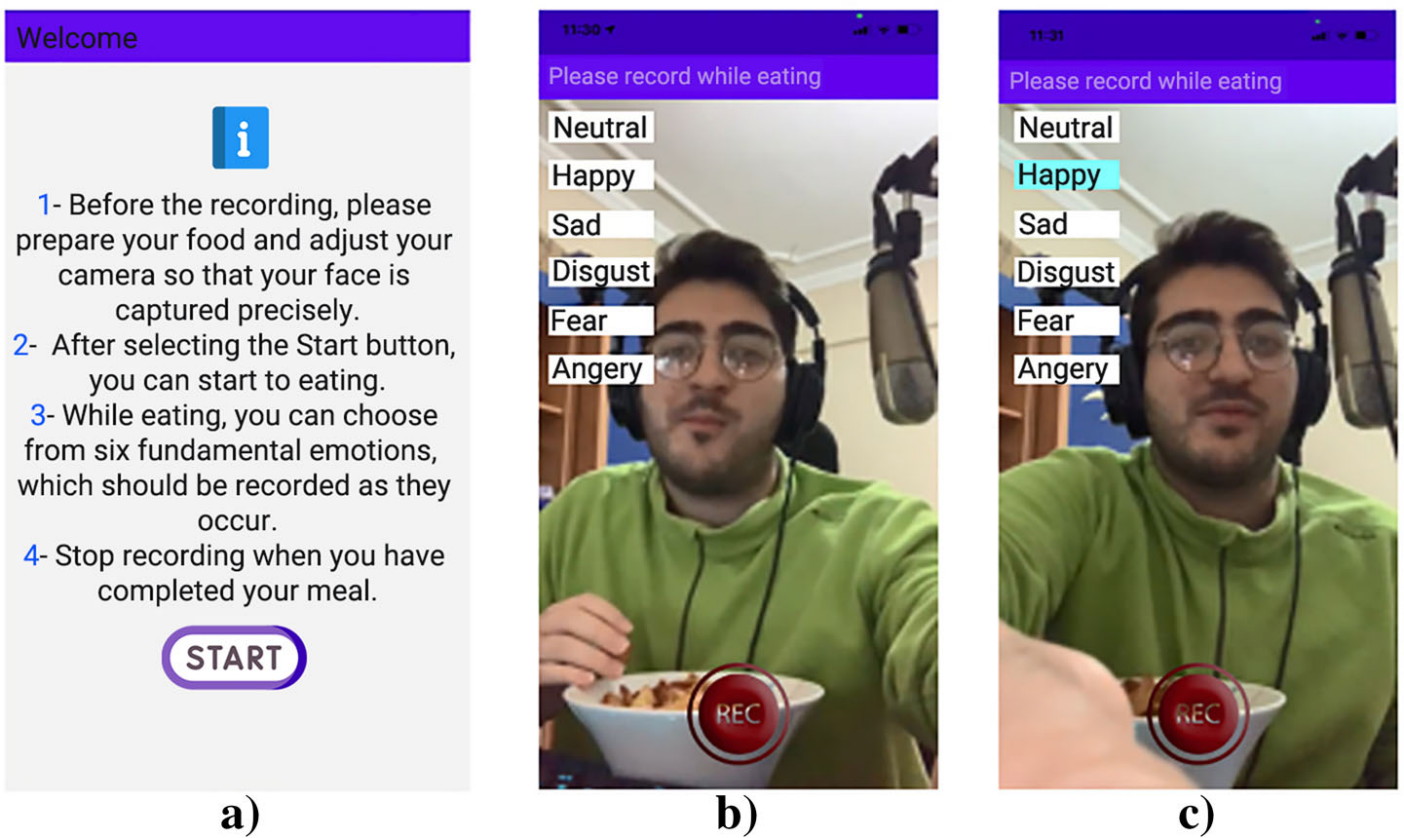
Sample screenshots of the mobile application to collect emotion during eating a) Landing screen that explains the instructions during the usage, b) Video is taken with the whole face visible during eating, and c) After having an idea about the food eaten, emotion is selected from the list

In order to collect emotion while eating, we developed a mobile application (Fig. 1) with React Native [27] to be compatible with both Android and iOS mobile operating systems. React Native is a framework developed by Facebook and allows the development of applications in a cross-platform manner based on React JavaScript. It has a proprietary language format called JSX.

The emotion collection method to be used in this study was selected as a result of an analysis of widely used methods in the literature. The most common methods are the Self-Assessment Manikin (SAM) [7], Expressing Emotions and Experiences (3E) [35], and Emocards [12], which are used to collect emotions before, during, or after the use of the tested system. However, as emotions are usually very short instants [5], trying to remember them even just after the action is not a practical application for individuals. As such, we have developed an application to be able to capture expressions of emotions instantly as they are experienced, it can be considered as an experience sampling method [24].

Common methods such as the SAM represent what has been deliberated for appraising the emotions awakened by static features, mostly physical characteristics or look-and-feel. Thus, it is assumed that the emotion of the individual does not change expeditiously. There can be shifts progressively, however, those are not remarkably fast. Nevertheless, when the priority is on assessing the emotional occasions during eating triggered by active interaction such as food with a mobile application, it is unavoidable that the sentiments can change rapidly even between extremes. Hence, collecting instant information about emotions during app use becomes critical not only for reducing the time lapse between emotional experiences but also for capturing very short instant emotions, which are followed by new emotional experiences during an interaction.

This approach merges the mobile application and the emotion gathering tool in the alike system. The device is utilized for the self-assessment of emotion during eating. There are six basic emotion options that are offered to participants, namely neutral, happy, sad, disgusted, scared, and angry. There are no time and usage restrictions. During the eating activity, it is preferred to use small buttons in a mobile context by selecting an expression status on the display. Parallel to the method in the literature like SAM and Emocards, this approach also pushes the person to choose the best probable option from a set of choices. The proposed approach also concerns the problem of selecting the most proper emotion, but now the preference is accomplished rather often and usually just after an interaction has occurred, which should effectively diminish the necessity for establishing multiple expressions at once.

### Proposed deep emotion recognition model

Since we intend to use computerized emotion recognition approaches, we are concerned with the precision of measurements of facial features to get emotions. Because emotions can be specified by facial expressions, it is reasonable to match a collection of action units (AUs) to emotions. Most individuals cannot arbitrarily restrain their emotional expressions. Extreme situations make detecting emotions more difficult and can be managed by examining muscle activity over time rather than just pictures. Within the scope of the study, we developed a framework to transform instant facial expressions into emotions.

**Fig. 2:**
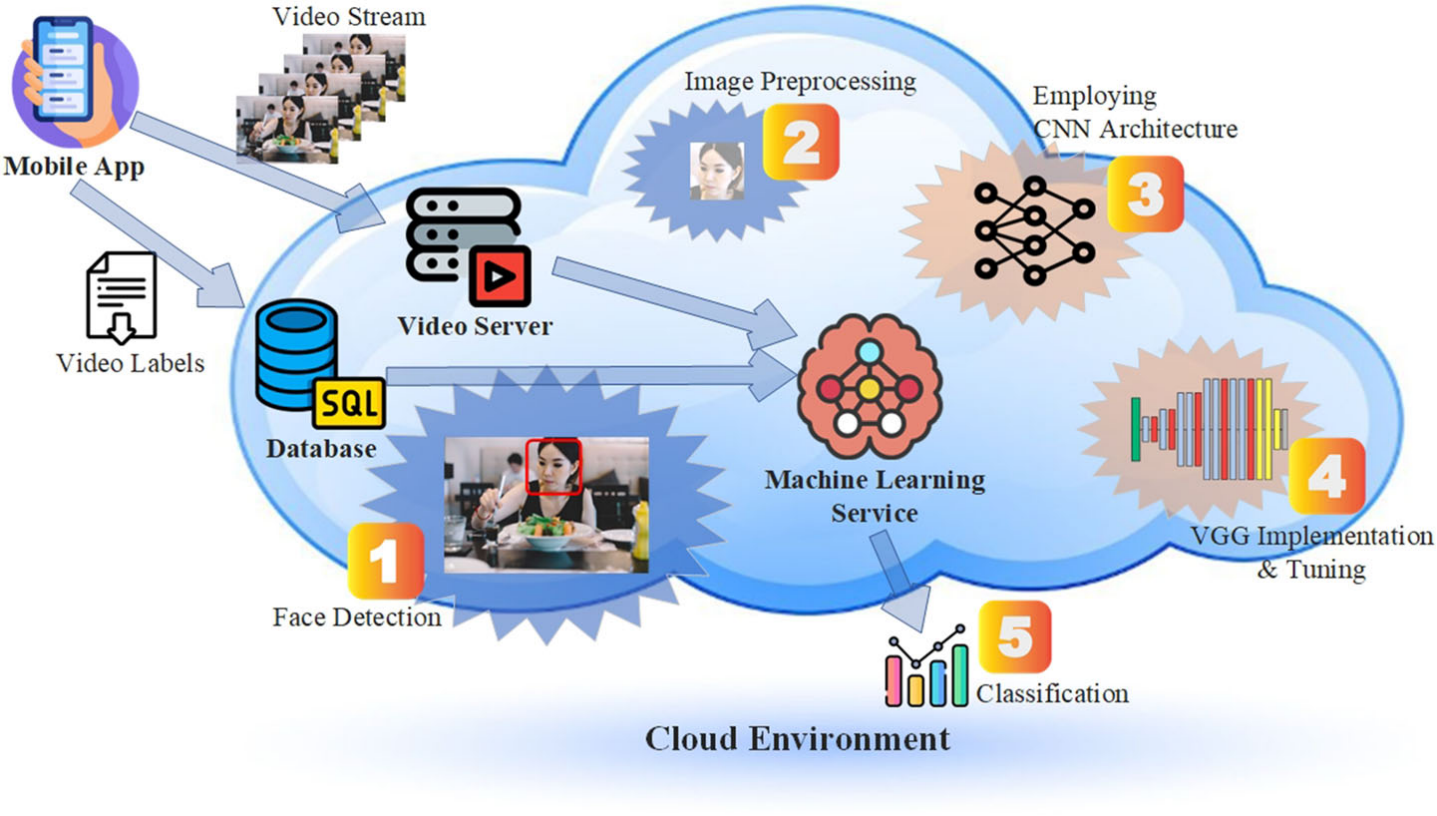
Implementation process of emotion recognition system

**Fig. 3:**
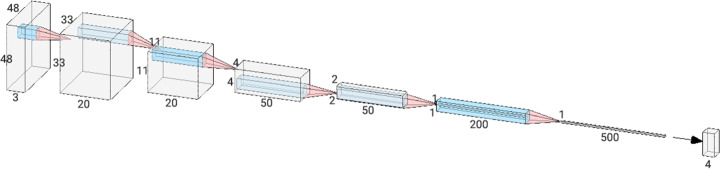
Architectural view of CNN model to predict four emotions

Figure 2 presents the outline of the developed prototypical implementation. The model follows a funnel logic to lower video frames to emotions and is divided into five main phases. Users collect facial data in video format using the mobile application’s camera. Later with phase 1, the process of detecting faces and removing facial features from video frames comes into play. Each detected face is determined and stored in the relevant frame, and then, the second stage processes begin. In the second phase, the data points are passed through an elimination process. In the beginning, each data points contain 20 frames that represent the same emotion, which is later reduced to 5 frames. The aim here is to select the most appropriate sequential frames to create sequential images. After detecting related frames, facial regions are detected by MTCNN [40] for all of the training and test samples. Then, five critical facial points such as the eyes, nose, and the corners of the mouth are used to perform the resemblance transformation. We obtain the cropped faces and resize them to be 48x48 pixels. Each pixel at [0, 255] in images is normalized. This input was used as phase 3 with a conventional CNN architecture. After the conventional CNN implementation was not successful, VGG16 was added and tuned (phase 4) and the results were calculated (phase 5). To perform classification we calculate the probability of each emotion with deep learning algorithms. The proposed emotion identification architecture extracts facial attributes that are related to various emotions and applies them as an additional modality to detect emotions. The architecture is built using two consecutive cascaded networks, which are depicted in Fig. 3.

The first network uses the VGG16 architecture with matching filter size, pooling, and convolutional layers. In the first layer, we applied 3×3 filters with a convolution stride of 1. Following the convolution operation, the main motivation was to keep spatial resolution, therefore the padding was fixed to 1 pixel. Max pooling was done on a 2 × 2 pixel window (i.e., stride is 2) and performed after each convolutional layer. One global average pooling that takes the average of each feature map obtained from the last convolutional layer was placed after the eleventh convolutional layer. As an activation function, we used Rectified Linear Units (ReLU) in each layer. We used Rectified Linear Units (ReLU) not only as an activation function in each hidden layer of the network but also as the prediction function at the last layer. In the study, we trained our model with public datasets such as FER-2013 [18] and CK+ [26] datasets and verified results using our collected samples via mobile application.

## Experimental results

In this section, we discuss the results of our CNN-based model, which is developed for emotion recognition from facial expressions. We analyzed and compared the effectiveness of a couple of deep learning methods. We chose our training set from public databases such as FER-2013 and CK+ and our test set was assembled with face images constructed via our mobile application during eating activity. The developed system is designed to work in real-time, therefore, the most accurate output is aimed.

Participants were seated in a standard home environment and initiated a video session via mobile application. What they need to do in this process is just to taste or see the food. The main task is to rate each participant’s feelings either tasting meals or seeing them and reporting via the mobile application. The camera of the mobile phone was used to capture video in real-time that contains various facial expressions corresponding to four emotional categories. The emotion captured was instantly reflected on the display. The association between listening to music and watching video content while eating with food consumption in the natural environment was assessed using 13 participants between the ages of 18 and 25. It was reported that four different emotions were felt during the experiment, they are neutral, happy, sad, and disgusted. We recorded while they ate their meal with three case studies such as alone eating, eating while listening to music, and watching audiovisual content.

Table 2 demonstrates the results of our CNN-based model on the gathered validation set. It can be noticed that fine-tuned architecture performs quite well in neutral and happy cases. However, the performance on sad and disgusted is average, mostly due to relatively fewer training samples.

**Table 2:** Average accuracy for each emotion

Method	Neutral	Happy	Sad	Disgusted	Overall accuracy
VGG16	79.36%	76.91%	64.34%	68.17%	72.20%
Fine tuned VGG16	84.14%	88.38%	68.67%	69.51%	77.68%
Deepface	81.53%	90.63%	62.13%	59.89%	73.54%

**Fig. 4:**
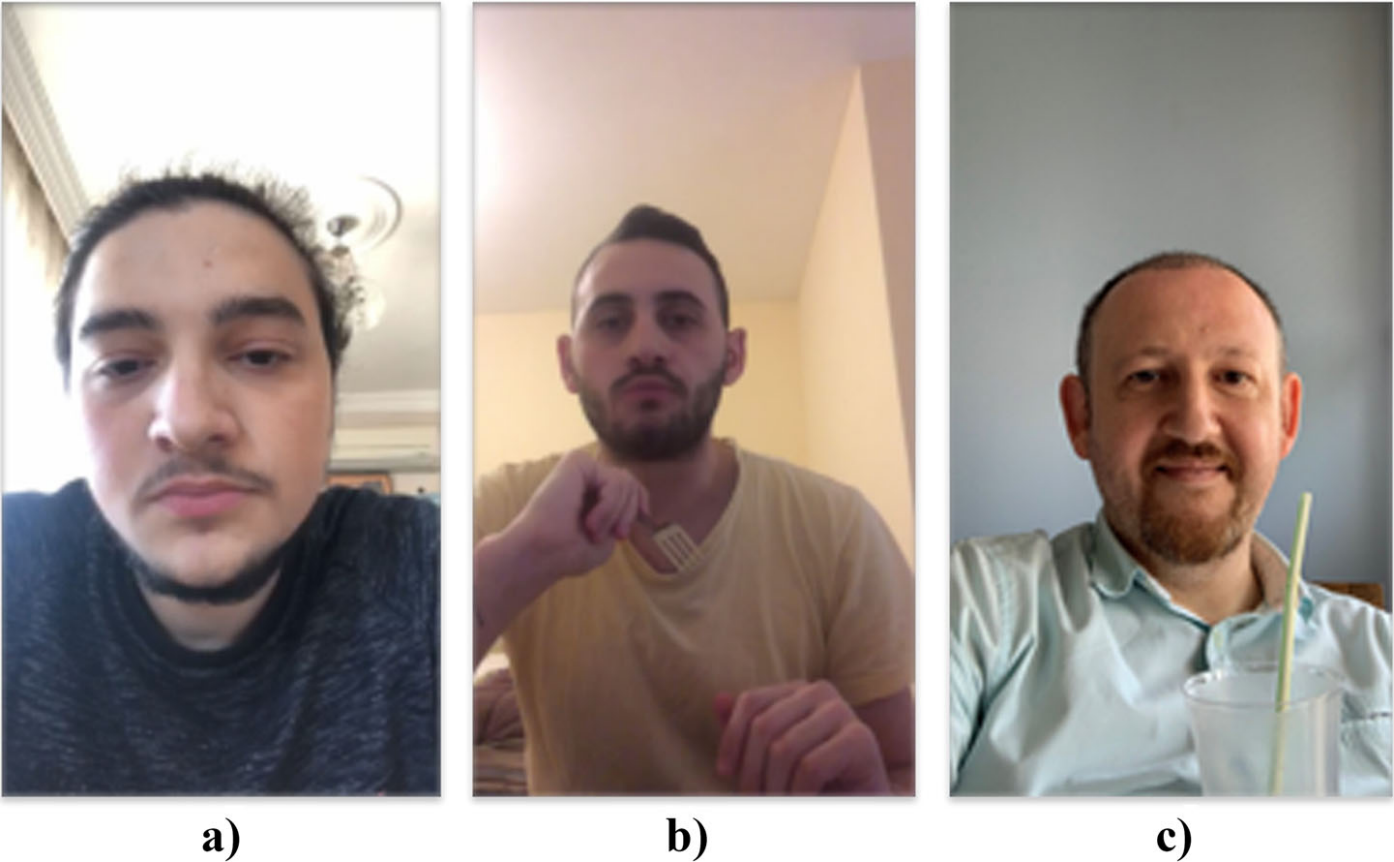
Sample facial expression during eating : a) the dominant emotion of the first image is ‘neutral’ with accuracy of 92.64% b) the second image displays ‘neutral’ emotion with accuracy of 63.69%, and c) third image exhibits the ‘happy’ emotion with accuracy of 92.71%

The gathered data show that the presence of music is associated with higher food intake. Within-participant comparisons exposed higher food intake and more extended meal periods while listening to music but no significant differences in music speed or volume. The existence of music seems to be not an effective method of changing the positive or negative emotional states that influences food intake. The sample view is shown in Fig. 4.

In Fig. 4a, the participant’s mood was neutral while eating alone without any environmental effect. In Fig. 4b, the participant started to listen to music, but his dominant emotion was still neutral. In Fig. 4c, his dominant emotion changed to ‘happy’ after he started to watch a video.

## Discussion

### The hypothesis

Our proposed system hypothesized the following statements;

1. Food reviews are better conducted with emotion detection using face recognition via mobile app.
2. Emotion recognition accuracy is higher in food review made in a musical environment than in a non-musical environment.
3. The employment of pretrained models with conventional methods brings higher performance in emotion recognition from facial expression.

In line with the hypothesis (i), we observed the data provided by automatic emotion recognition systems eliminates the user’s deception factor. Detecting the tendency of users to choose inaccurate answers in paper-based surveys is often ignored as a difficult problem. The absence of this situation in the proposed system enables it to be used as a more effective approach.

We also found that the classification accuracy of reviews made in music playing environments was on average 3.54% higher as hypothesis (ii) association. Especially happy as the most affected emotion with 14.6% shows the effect of musical environments on the selection of positive emotions. These results showed that emotions were experienced more intensely in musical environments. Our experiments support the hypothesis (iii) as, the results in Table 2 show the effectiveness of pretrained models, as successful results cannot be obtained with the conventional CNN approach.

### Threats to validity

Since the proposed system is affected by various factors, we identified the possible threats in the perspective of internal and external validity. As the first threat of internal validity, participant selection bias affects the results. It is known that obese people experience higher positive emotions when eating than average people [17]. For this reason, body mass index (BMI) should be considered as an important indicator in the selection of participants. Maturation should be considered as the second factor. Eating assessments are not legitimate for older people, mostly due to progressive age-related health problems.

As the external validity perspective we identified situational factors such as facial expressions cannot be extracted from the video taken due to the use of the mobile application in a very dark or very bright environment. Similarly, the face must be seen from the front during video shooting. If the participant uploads a video shot from the side that only shows part of his face, the system will not be successful. If there is more than one face during the review, an undesirable situation occurs that negatively affects the emotion analysis. Finally, there may be users who cannot get the use of the mobile app. There are instructions on the opening interface for this, and the development was conducted by considering the human–computer interaction (HCI) and user experience (UX) design principles.

## Conclusion

The relationship between emotion and eating is a topic that has been being studied for a long time and its origin is based on the obesity literature. Therefore, previous studies have mostly focused on explaining eating in obese individuals, however, new theories aim to explain the eating behavior of people of normal weight. Thus, our research focus is superintended on the emotional dimension of eating occasions. Emotions vary in duration and frequency of sensation, trigger patterns and locations, and physiological correlates. Relationships between a certain emotion and eating behavior should be stronger if it occurs more frequently than other emotions during eating.

While we were aiming for a method to recognize basic emotions such as happiness, disgust, sadness, and others, studies show that there is no system that has achieved a perfect success rate yet. The reason is that the face can express many emotions at the same time. The systems used also make some assumptions based on facial expressions and gestures, therefore, the possibility of making mistakes increases in this emotional intensity.

During our empirical experiments, we have employed various generic deep learning algorithms as well as pre-trained models such as VGG16. We observed that fine-tuned VGG16 performed quite well in neutral and happy cases. This model achieved an overall accuracy of 77.68%, while Deepface resulted with 73.54% and traditional VGG16 was 72.20%. We concluded that supportive studies are needed to recognize disgust and sad emotions for all deep models. We plan to add new functionalities and evaluate other deep learning-based models to improve the overall accuracy. We manually asked the users to feedback on the emotions they felt at the moment, which was used to validate the predictions of the system. This study can be used in all research on the relationship between eating and emotion. For example, in understanding the feelings of eating disorders and depression. Similarly, it can pave the way for applications that disable the human factor in food evaluations. More realistic results eliminate survey-based assessments with successful implementations.

## Data Availability

The datasets generated during and/or analysed during the current study are available from the corresponding author on reasonable request.
